# Combined anticancer activity of osthole and cisplatin in NCI-H460 lung cancer cells *in vitro*

**DOI:** 10.3892/etm.2013.889

**Published:** 2013-01-08

**Authors:** XIAO-MAN XU, YI ZHANG, DAN QU, HONG-BO LIU, XIU GU, GUANG-YU JIAO, LI ZHAO

**Affiliations:** 1 Department of Respiratory Medicine, Shengjing Hospital of China Medical University, Shenyang, Liaoning 110004, P.R. China; 2 Department of Geriatrics, Shengjing Hospital of China Medical University, Shenyang, Liaoning 110004, P.R. China

**Keywords:** osthole, cisplatin, NCI-H460 cells, apoptosis

## Abstract

Drug combination therapies are common practice in the treatment of cancer. Cisplatin is the most active chemotherapeutic agent for lung cancer treatment. Osthole is a natural compound extracted from a number of medicinal plants. To determine whether osthole enhances the anticancer effect of cisplatin in human lung cancer, we treated NCI-H460 cells with osthole alone or in combination with cisplatin and evaluated cell growth and apoptosis using 3-(4,5-dimethyl thiazol-2yl)-2,5-diphenyltetrazolium bromide (MTT) assay, flow cytometry and fluorescence microscopy. The results showed that, in comparison with single agent treatment, the combination of osthole and cisplatin resulted in greater efficacy in growth inhibition and apoptosis induction. Western blot analysis revealed that the combination effect of osthole and cisplatin was due to regulation of the Bcl-2 family proteins. Findings of this investigation suggested that osthole combined with cisplatin is a potential clinical chemotherapeutic approach in human lung cancer.

## Introduction

Lung cancer remains the most common human cancer in the worldwide, with non-small cell lung cancer (NSCLC) accounting for ∼80% of cases (1,2). Despite great achievements made over the past decades in surgery, radiotherapy and chemotherapy, the 5-year survival rate of lung cancer in many countries is <15% (3). Chemotherapy remains the mainstay of treatments of lung cancer and cisplatin is one of the most widely used first-line chemotherapeutic agents for NSCLC treatment (4). However, these therapeutic strategies are unsatisfactory due to side effects experienced and drug resistance. Thus, identification of new therapeutic agents that exert combination effects with cisplatin for the treatment of NSCLC is necessary.

**Figure 1.**
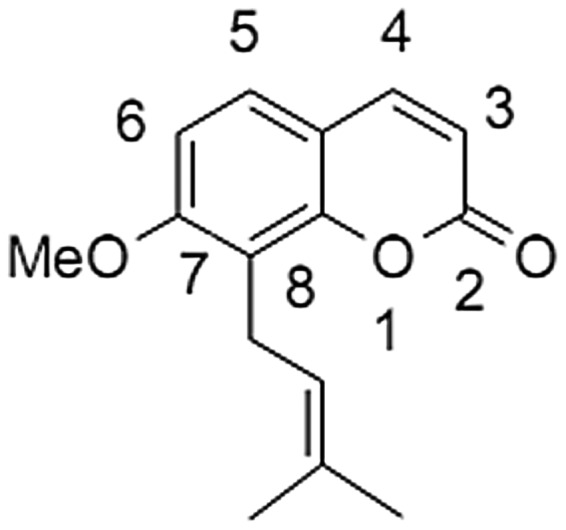
The structure of osthole.

Osthole (Fig. 1), a natural compound, is extracted from various medicinal plants such as *Cnidium monnieri* (L.) *Cusson*. In recent years, numerous studies have focused on the potential of osthole in cancer therapy. Osthole reportedly possesses anticancer effects by inhibiting cancer cell growth, metastasis and inducing cell apoptosis (5–9). Recently, we reported that osthole induces G2/M arrest and apoptosis by modulating the PI3K/Akt pathway and suppresses migration and invasion through inhibition of matrix metalloproteinase-2 and matrix metallopeptidase-9 in human lung adenocarcinoma A549 cells (10,11). However, the combination effects of osthole and cisplatin on human lung cancer cells remain unclear.

The aim of the present study was to investigate the anti-cancer activity of combining osthole with cisplatin in human large cell lung carcinoma NCI-H460 cells *in vitro*. The results demonstrated that the combination of osthole and cisplatin resulted in greater efficacy in growth inhibition and apoptosis induction in NCI-H460 lung cancer cells.

## Materials and methods

### Reagents and chemicals

Osthole and cisplatin were purchased from the National Institute for the control of Pharmaceutical and Biological Products (Beijing, China). Cell culture RPMI-1640 medium, antibiotics and trypsin were purchased from Biological Industries (Kibutz Beit Haemek, Israel). Fetal bovine serum (FBS) was purchased from Solarbio Science & Technology Co., Ltd. (Beijing, China). 3-(4,5-dimethyl thiazol-2yl)-2,5-diphenyltetrazolium bromide (MTT), dimethyl sulfoxide (DMSO) and Hoechst 33342 were purchased from Sigma-Aldrich (St. Louis, MO, USA). Annexin V-FITC and PI double staining kit were purchased from Key Gene (Nanjing, China). Antibodies were purchased from Santa Cruz Biotechnology, Inc. (Santa Cruz, CA, USA). The other reagents were procured locally.

### Cell culture

Human large cell lung carcinoma NCI-H460 cells were obtained from the China Center for Type Culture Collection (Wuhan, China) and maintained in RPMI-1640 supplemented with 10% FBS, 100 U/ml penicillin and 100 *μ*g/ml streptomycin at 37°C in a humidified atmosphere of 5% CO_2_.

### Cell viability assay

The effect of osthole, cisplatin and their combination on the proliferation of NCI-H460 cells was measured by MTT assay. Briefly, NCI-H460 cells were plated at a density of 1×10^4^ cells per well in 96-well plates overnight and then treated with 0, 50, 100 and 200 *μ*mol/l osthole; 1.5, 3.0 and 6.0 *μ*mol/l cisplatin or their combination (50 *μ*mol/l osthole plus 1.5 *μ*mol/l cisplatin ) for 48 h. Twenty microliters of MTT solution (2 mg/ml in PBS) were added to each well and the cells were cultured for another 4 h at 37°C. The medium was completely removed and 150 *μ*l DMSO was added to solubilize MTT formazan crystals. The plates were then agitated and the optical density was determined at 570 nm (OD570) using an ELISA plate reader (Model 550; Bio-Rad, Hercules, CA, USA). At least three independent experiments were performed.

### Annexin V/PI flow cytometric analysis

Apoptotic rates were determined by flow cytometry using an Annexin V-FITC apoptosis kit. Briefly, NCI-H460 cells were seeded at a density of 1×10^6^ cells per well in 6-well plates overnight and then treated with 50 *μ*mol/l osthole, 1.5 *μ*mol/l cisplatin or their combination for 48 h. Cells (1×10^6^) were collected by centrifugation (326 × g) and washed twice with cold PBS. Staining was performed according to the manufacturer’s instructions and the cells were analyzed using a FACScan flow cytometer and analyzed using CellQuest software (Becton-Dickinson, Redlands, CA, USA). At least three independent experiments were performed.

### Fluorescence microscopy

NCI-H460 cells (1×10^6^) were seeded in 6-well plates overnight and then treated with 50 *μ*mol/l osthole, 1.5 *μ*mol/l cisplatin or their combination for 48 h. Cells were washed twice with PBS and fixed with cold methanol and acetic acid (3/1, v/v) overnight, then stained with Hoechst 33342 (1 mg/ml) for 30 min in the dark. Stained cells were observed with a fluorescence microscope (magnification, ×400) (Nikon, Tokyo, Japan).

### Western blot analysis

NCI-H460 cells (1×10^6^) were seeded in 6-well plates overnight. Cells were treated as described above. Cells were then washed twice with ice-cold PBS. The total proteins were solubilized and extracted with lysis buffer (20 mM HEPES, pH 7.9, 20% glycerol, 200 mM KCl, 0.5 mM EDTA, 0.5% NP40, 0.5 mM DTT, 1% protease inhibitor cocktail). Protein concentration was determined by bicinchoninic acid protein assay. The samples were separated by SDS-PAGE, transferred to PVDF membranes by electroblotting and probed with anti-Bax, anti-Bcl-2 and anti-actin. Membranes were then incubated with horseradish peroxidase-conjugated secondary antibodies. Blots were developed using an ECL kit.

### Statistical analysis

The experiments were performed for three times. Data were expressed as the mean ± standard deviation (SD). Statistical correlation of data was checked for significance by ANOVA and Student’s t test. P<0.05 was considered to indicate a statistically significant difference. These analyses were performed using SPSS 13.0 software.

## Results

### Combined effect of osthole and cisplatin on NCI-H460 cell viability

**Figure 2.**
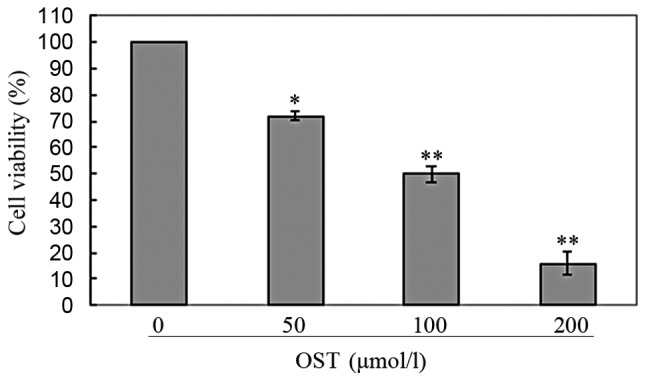
The proliferative inhibitory effects of osthole (OST) on NCI-H460 cells. ^*^P<0.05 vs. control group. ^**^P<0.01 vs. control group.

**Figure 3.**
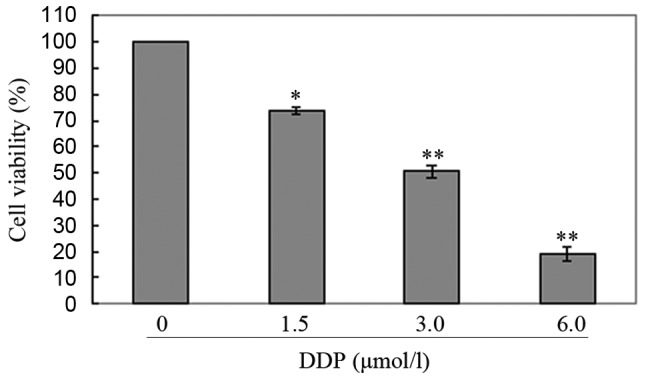
The proliferative inhibitory effects of cisplatin (DDP) on NCIH460 cells. ^*^P<0.05 vs. control group. ^**^P<0.01 vs. control group.

**Figure 4.**
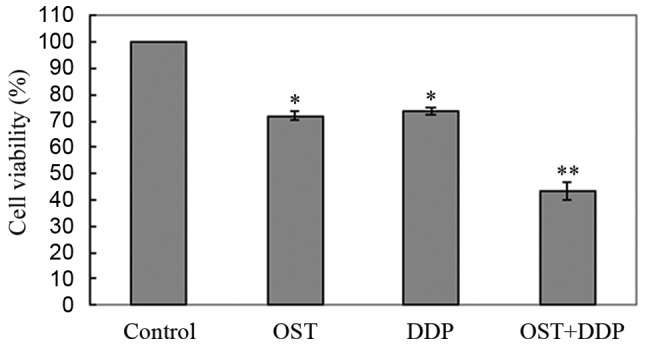
Combined effect of osthole (OST) and cisplatin (DDP) on NCI-H460 cell proliferation. ^*^P<0.05 vs. control group. ^**^P<0.01 vs. control group.

To investigate the combined effect of osthole and cisplatin on the cell viability of NCI-H460 lung cancer cells, the cells were treated with various doses of osthole and cisplatin for 48 h, respectively. Cell viability was determined by MTT assay. It was noted that both osthole (Fig. 2) and cisplatin (Fig. 3) inhibited cell proliferation in a dose-dependent manner. Based on these results, we selected a moderate concentration (50 *μ*mol/l osthole plus 1.5 *μ*mol/l cisplatin) for combination treatment. Compared with monotherapy, combination treatment inhibited cell proliferation more significantly (Fig. 4).

### Combined effect of osthole and cisplatin on NCI-H460 cell apoptosis

**Figure 5.**
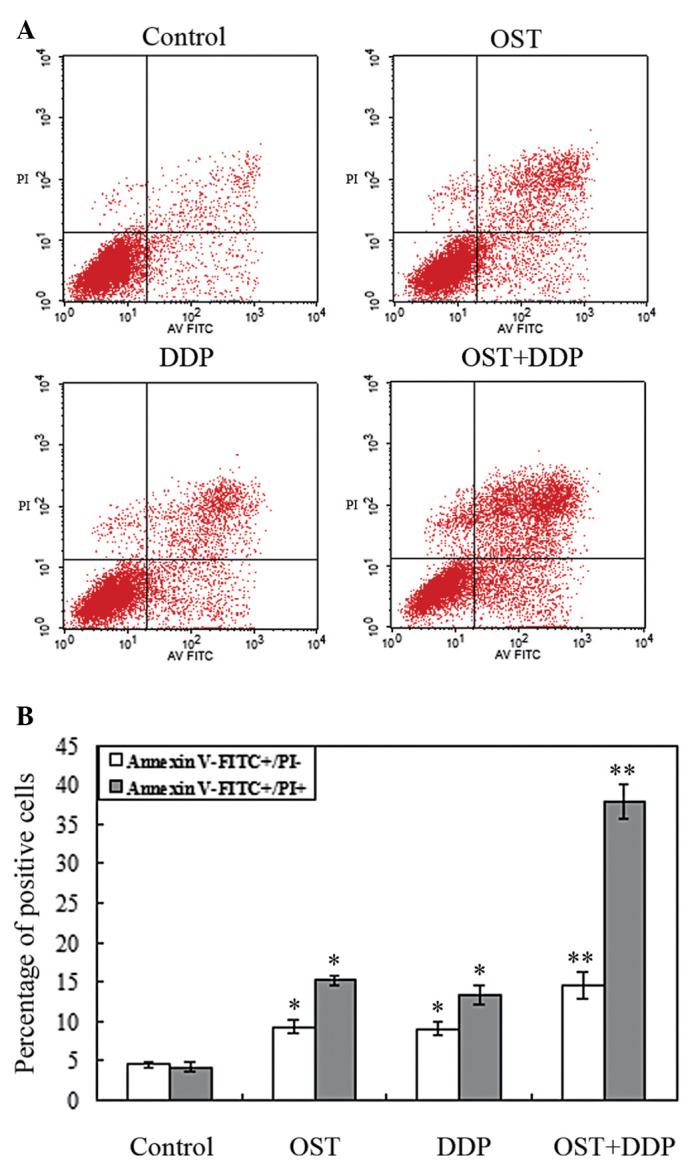
Combined effect of osthole (OST) and cisplatin (DDP) on NCI-H460 cell apoptosis. (A and B) Osthole or cisplatin alone induced NCI-H460 cell apoptosis. The percentage of early and late apoptotic cells was increased compared to the control group. However, compared with monotherapy, the percentage of apoptotic cells induced by their combination was significantly higher. (B) Summaries of the apoptosis data in histograms.^*^P<0.05 vs. control group; ^**^P<0.01 vs. control group.

The apoptosis-inducing effect of osthole, cisplatin and their combination was evaluated by Annexin V/PI staining. NCI-H460 cells were treated with 50 *μ*mol/l osthole, 1.5 *μ*mol/l cisplatin or their combination for 48 h and were analyzed by flow cytometry to analyze apoptosis. As shown in Fig. 5A and B, osthole or cisplatin alone induced NCI-H460 cell apoptosis. The percentage of early and late apoptotic cells was increased compared to the control group.

Compared with monotherapy, the percentage of apoptotic cells induced by their combination was significantly higher.

**Figure 6.**
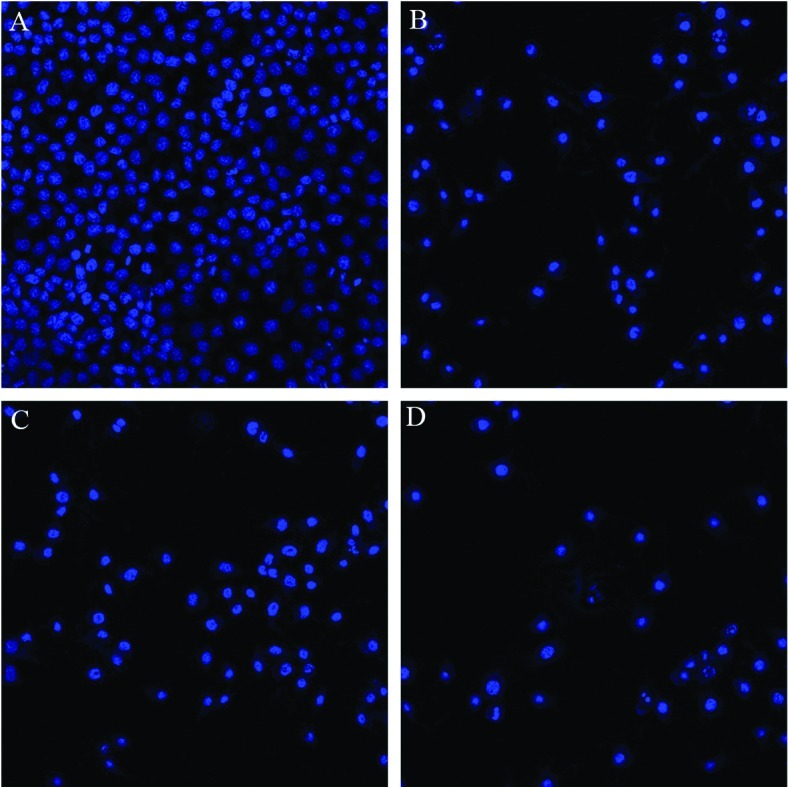
Cell apoptosis observed by Hoechst 33342 staining. NCI-H460 cells were treated with (B) 50 *μ*mol/l osthole (OST), (C) 1.5 *μ*mol/l cisplatin (DDP) or (D) their combination for 48 h. (A) Compared with the control group, treated cells exhibited chromatin condensation, nuclear fragmentation and apoptotic bodies.

Following incubation with 50 *μ*mol/l osthole, 1.5 *μ*mol/l cisplatin or their combination for 48 h, the cells were examined by fluorescence microscopy. Condensation of chromatin, nuclear fragmentations and apoptotic bodies were clearly identified in treated cells (Fig. 6). Compared with monotherapy, apoptotic cells significantly increased in the combination treatment.

### Combined effect of osthole and cisplatin on the expression of Bcl-2 family proteins

**Figure 7.**
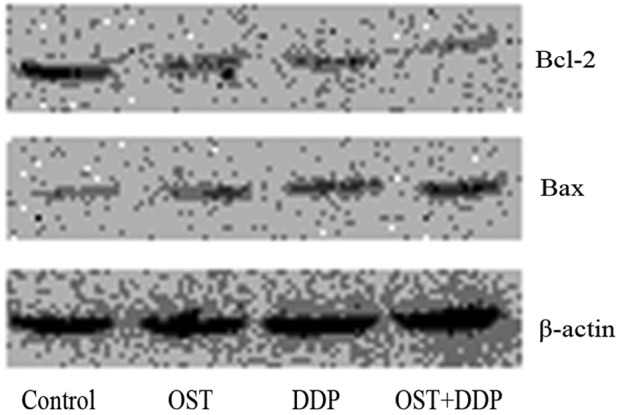
Combined effect of osthole (OST) and cisplatin (DDP) on expression of Bcl-2 family proteins. NCI-H460 cells were treated with 50 *μ*mol/l osthole (OST), 1.5 *μ*mol/l cisplatin (DDP) or their combination for 48 h. Proteins were extracted, and the expression of Bax, Bcl-2 and β-actin was analyzed by western blot analysis.

To investigate the molecular mechanism of the combined anticancer effect of osthole with cisplatin, we tested the effect of osthole, cisplatin or their combination on the expression of Bcl-2 and Bax. As shown in Fig. 7, western blot analysis revealed that treatment with either osthole or cisplatin decreased Bcl-2 expression and increased Bax expression. Compared with monotherapy, combination treatment with osthole and cisplatin decreased Bcl-2 expression and increased Bax expression more significantly.

## Discussion

Drug combination therapies are common practice in the treatment of cancer. At present, cisplatin is the most active chemotherapeutic agent for the treatment of NSCLC and is usually combined with other agents such as docetaxel, gemcitabine and paclitaxel (4). However, its use is limited due to severe side effects such as anemia, neurotoxicity, nephrotoxicity and the acquisition of drug resistance (12,13). To address these problems, attention has been focused on identifying novel agents that can be combined with cisplatin to increase the therapeutic efficacy and decrease side effects.

Recently, several studies reported that some natural agents combined with cisplatin enhanced the anticancer effects of various types of cancer (14–18). The combinatorial use of conventional chemotherapeutic agents and these natural compounds exert enhanced anticancer activity through combination effects. Combination treatment may also decrease the side effects since efficacy can be achieved with lower doses. In the present study, we have studied the effect of combining osthole, a natural compound extracted from various medicinal plants, with cisplatin on NCI-H460 lung cancer cells.

The mechanism of action of cisplatin includes the inhibition of cell proliferation and induction of cell apoptosis (19). To determine whether osthole enhances the anticancer effect of cisplatin, NCI-H460 cells were treated with osthole alone or in combination with cisplatin and cell growth and apoptosis were evaluated using MTT assay, flow cytometry assay and fluorescence microscopy. We found that in comparison with single agent treatment, the combination of osthole and cisplatin produced greater efficacy in growth inhibition and apoptosis induction, suggesting that osthole plays a combination role in cisplatin-induced apoptosis and growth inhibition in lung cancer cells.

Bcl-2 family proteins are key regulators of the apoptotic pathway (20). To elucidate the molecular mechanisms of the combined anticancer effect of osthole with cisplatin, we investigated the effects of osthole alone and in combination with cisplatin on Bcl-2 family proteins in NCI-H460 cells using western blot analysis. Our results showed that treatment of NCI-H460 cells with osthole in combination with cisplatin significantly decreased Bcl-2 expression and increased Bax expression, indicating that these agents induced apoptosis through regulation of the expression of Bcl-2 family proteins.

In conclusion, findings of the present study have shown that osthole enhanced the anticancer effect of cisplatin on human lung cancer cells *in vitro*. This investigation suggests that the combination of osthole and cisplatin may serve as an important potential clinical chemotherapeutic approach in human lung cancer.
